# An Acute Case of Herpes Zoster Ophthalmicus with Ophthalmoplegia

**DOI:** 10.1155/2012/953910

**Published:** 2012-05-09

**Authors:** Wasim Hakim, Rosalie Sherman, Tamer Rezk, Kanwar Pannu

**Affiliations:** ^1^The Postgraduate Education Centre, Basildon University Hospital, Essex SS16 5NL, UK; ^2^Department of Respiratory Medicine, The London Chest Hospital, Bonner Road, London E2 9JX, UK

## Abstract

Herpes zoster ophthalmicus (HZO) with oculomotor nerve involvement is rare, even rarer as an acute presentation rather than sequelae of HZO. In this paper we present a case of cutaneous HZO in which our patient's initial presentation was one of complete ophthalmoplegia.

## 1. Introduction

Herpes zoster (or shingles) refers to a typically vesicular rash caused by reactivation of the latent varicella zoster virus (chickenpox) from the dorsal root ganglia neurons. It usually presents in thoracic or cranial dermatoms. The lifetime risk of herpes zoster is estimated to be 10% to 20%, but in patients over the age of 80 years this risk rises to 50%. Reactivation can occur for a number of different reasons including trauma, ageing, or immune deficiency [1].

HZO is a rare form of shingles, reported in 15–25% of cases [2], that presents with a rash in the distribution of the trigeminal nerve dermatomes (mainly ophthalmic and maxillary divisions). It is often reported to be associated with a variety of complications, including episcleritis, keratitis, glaucoma, and cataracts [1], but there are very few reports of complete ophthalmoplegia being one of those.

## 2. Case Presentation

An 87-year-old lady who lives alone presented to the medical admissions unit with an inability to open her right eye. She has a past history of mild dementia and depression and was previous to this otherwise fit and well. She describes a 6-day history of blister formation surrounding the eye that extends to her right forehead and scalp. Her family noticed her eye was becoming increasingly droopy, red, and swollen culminating in it being permanently shut for twenty-four hours prior to admission. This visual impairment was most likely responsible for her falling before attending hospital, during which she sustained a left elbow laceration. Of note, she had been started two days previously on flucloxacillin and phenoxymethylpenicillin by her general practitioner.

**Figure 1:**
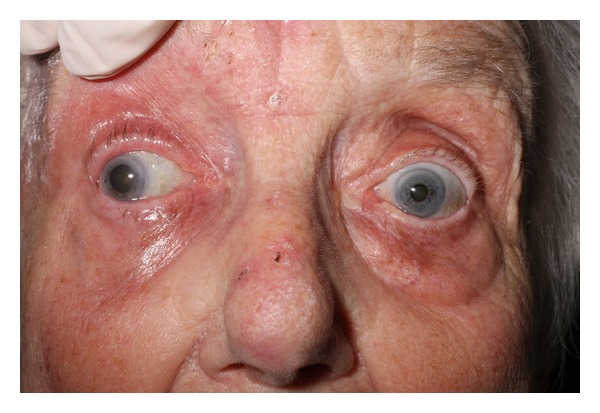
Oculomotor nerve palsy-pupil fixed and dilated in a position of lateral and downward gaze.

**Figure 2:**
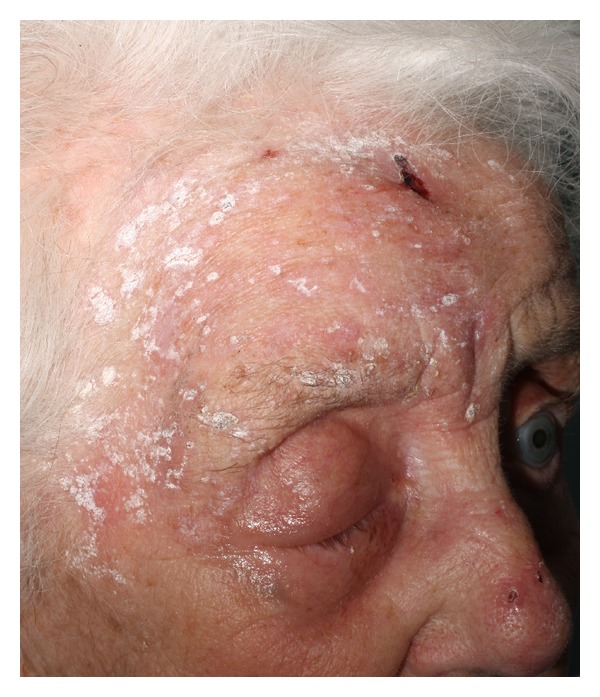
To show the rash in the distribution of the ophthalmic branch of the trigeminal nerve.

On examination she had a vesicular rash covering her right scalp, forehead, eye, and upper cheek. It was erythematous, swollen, and tender. She had a complete ophthalmoplegia, and her pupil was fixed and dilated. Her visual acuity in that eye was reduced to counting fingers. The remainder of her neurological and other systems examinations were normal. She was commenced on oral and topical acyclovir, dexamethasone, and cyclopentolate subsequent to ophthalmology review. There was no evidence of vasculitis on slit-lamp examination (see Figures 1 and 2).

The vesicular rash resolved some two weeks later after admission, and she was discharged. She had regained some of her eye movements partially but the ptosis remained.

## 3. Discussion

In the limited literature, that does report opthalmoplegia as part of the sequelae of HZO, this has typically been described as a late complication, often up to 2 months after the initial herpetic rash [3] and is seen in only 11–29% of patients with HZO. However, in our case it has developed as part of the acute viral infection. The most commonly affected nerve is the third cranial nerve and, less commonly, the fourth nerve [4]. With a third nerve palsy it has been reported as being partial or complete but there is always ptosis. Isolated ptosis and isolated paralysis of the pupil have also been seen [5]. In our case, there was third cranial nerve (oculomotor) palsy causing complete external ophthalmoplegia, with her pupil fixed and middilated.

Several hypotheses have been proposed for the mechanism behind which HZO can result in ophthalmoplegia [5–8] although it is most likely that there are many contributing factors. It is known that the reactivated virus causes inflammation of the axons that supply the dermatomes in question. Edgerton suggested that the inflammation of the trigeminal nerve could actually spread via the cavernous sinus to affect the oculomotor nerve [5]. In addition, Naumann et al. found chronic inflammatory cells suggesting an occlusive vasculitis [6]. Carrol thought that due to the onset and rate of recovery, this pathology would suggest a demyelinating disease, and Lavin et al. were in agreement with this hypothesis based on autopsy reports [7, 8].

Diagnosis of this condition is essentially a clinical one based on history and examination findings. Once the diagnosis is made, it is reported that the earlier treatment is initiated, the better the prognosis is, preferably within 72 hours, although beneficial effects have been reported with treatment started as late as 7 days after onset [9]. Acyclovir, famciclovir or valacyclovir have all been used, and they act by resolving skin lesions, decreasing viral shedding and decreasing the risk of ocular involvement. Some report that famciclovir and valacyclovir are better at resolving pain associated with HZO than acyclovir [9]. In addition to the antivirals it is important to treat any further complications detected. For example, if keratitis or episcleritis has developed, then topical steroids can be used. The effectiveness of steroids and antivirals alone or in combination does not, however, appear to have been formally studied, and treatment options are limited partly due to the poorly defined mechanism of HZO.

The prognosis for full recovery after complete opthalmoplegia following HZO is good. In one literature review, of 16 total cases, 9 were followed up, and all of those cases showed significant improvement in symptoms after 2 months and almost complete resolution by 18 months [10].

## 4. Conclusion

Ophthalmoplegia is a rare complication of HZO. Furthermore, the cases in the literature describe it as relatively late sequelae, unlike the acute presentation we have reported.

## Abbreviation

HZO: Herpes zoster ophthalmicus.
